# Chronic kernicterus: magnetic resonance imaging findings

**DOI:** 10.1590/0100-3984.2015.0190

**Published:** 2016

**Authors:** Bruno Niemeyer de Freitas Ribeiro, Gabriela de Almeida Lima, Nina Ventura, Emerson Leandro Gasparetto, Edson Marchiori

**Affiliations:** 1 Instituto Estadual do Cérebro Paulo Niemeyer, Rio de Janeiro, RJ, Brazil.; 2 Universidade Federal do Rio de Janeiro (UFRJ), Rio de Janeiro, RJ, Brazil.

**Figure 1:**
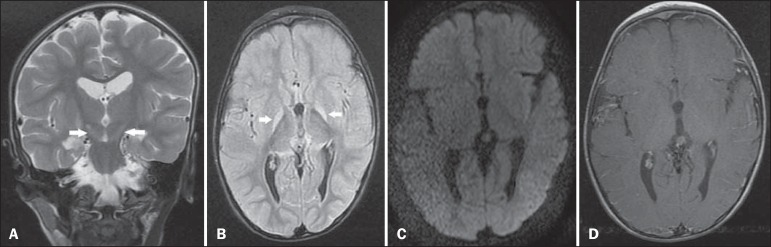
**A:** Coronal T2-weighted MRI sequence showing a bilateral, symmetrical hyperintense signal in the subthalamic nuclei (arrows), without a mass effect. **B:** Axial FLAIR MRI sequence showing a bilateral, symmetrical hyperintense signal in the globus pallidus (arrows). **C:** Axial diffusion-weighted MRI sequence showing no diffusion restriction. **D:** Axial T1-weighted MRI sequence showing no evidence of gadolinium enhancement.

Dear Editor,

A 3-year-old male child who had developed bilirubin encephalopathy in the neonatal period, due to Rh incompatibility, presented with delayed neuromotor/psychomotor development and involuntary movements. The prenatal and perinatal periods had been free of complications. Serology for cytomegalovirus, toxoplasmosis, and HIV were negative, as was the VDRL test. The results of a complete blood count, serum ceruloplasmin, electrolytes, and thyroid function were all within the limits of normality. Magnetic resonance imaging (MRI) of the brain showed bilateral, symmetrical hyperintense signals on FLAIR and T2-weighted sequences, affecting the globus pallidus and subthalamic nuclei, with no mass effect, with no diffusion restriction or evidence of gadolinium enhancement (Figure 1). Those imaging findings, together with the clinical and biochemical history, confirmed the suspected diagnosis of chronic kernicterus.

Recent studies conducted in Brazil have highlighted the importance of MRI studies to improving the diagnosis of central nervous system disorders^(1-5)^. Kernicterus, also known as bilirubin encephalopathy, is a rare complication of hyperbilirubinemia in childhood, occurring when serum bilirubin levels in the neonate are in excess of 20 mg/dL at term or even lower values in premature infants, which result in bilirubin deposition in the globus pallidus, subthalamic nuclei, hippocampus, putamen, thalamus, and cranial nerves, primarily the third, fourth, and sixth cranial nerves^(6)^. Symptoms include drowsiness, hypotonia, opisthotonus, rigidity, and seizures. The factors involved in its pathogenesis are hyperbilirubinemia, reduced serum bilirubin binding capacity, changes in the permeability of blood-brain barrier, and neurotoxicity. Although the main causes of kernicterus are ABO and Rh mismatches, it can also be caused by sepsis and other types of hemolytic anemia such as glucose-6-phosphate dehydrogenase deficiency^(7)^. The clinical symptoms and signs can regress completely if properly treated with phototherapy and blood transfusions^(6)^; without treatment, permanent damage can occur, generating encephalopathy with symptoms related to the basal nuclei, including involuntary movements, asymmetric spasticity, rigidity, ataxia, and hearing loss^(8)^.

The MRI findings in kernicterus are characterized by a hyperintense signal on T1-weighted sequences in the globus pallidus, progressing chronically to a shift from a hyperintense signal on T1-weighted sequences to a bilateral, symmetrical hyperintense signal on T2-weighted and FLAIR sequences in the globus pallidus and subthalamic nuclei^(7,9-11)^, corresponding to the areas of preferential deposition of unconjugated bilirubin, characterizing chronic kernicterus, as in the case presented here.

There is a broad spectrum of diagnoses of bilateral lesions in the basal ganglia in the pediatric population. The main causes cited are hypoxic-ischemic encephalopathy; hypoglycemia; encephalitis; inborn errors of metabolism; water and electrolyte disturbances; carbon monoxide poisoning; and demyelinating disorders. The correlation with clinical and laboratory data is fundamental for making the definitive diagnosis^(7,12,13)^.

In conclusion, the possibility of acute or chronic kernicterus should be considered when clinical symptoms, biochemical data, and MRI findings are suggestive of the disease, the chronic presentation and permanent, irreversible profile being promoted by bilirubin neurotoxicity.
